# CD25 as a unique marker on human basophils in stable-mildly symptomatic allergic asthma

**DOI:** 10.3389/fimmu.2022.1031268

**Published:** 2023-01-05

**Authors:** Joseena Iype, Lionel Rohner, Sofia Bachmann, Tanja Rahel Hermann, Nikolay Pavlov, Christophe von Garnier, Michaela Fux

**Affiliations:** ^1^ University Institute of Clinical Chemistry, Inselspital, Bern University Hospital, Bern, Switzerland; ^2^ Department of Pulmonary Medicine, Inselspital, Bern University Hospital, University of Bern, Bern, Switzerland; ^3^ Institute of Social and Preventive Medicine, University of Bern, Bern, Switzerland

**Keywords:** CD25, basophils, immunophenotype, stable-mildly symptomatic allergic asthma, *ex vivo* stimulation

## Abstract

**Background:**

Basophils in acute asthma exacerbation are activated as evidenced by their increased expression levels of activation markers such as CD203c and CD63. However, whether basophils of allergic asthmatics who are in stable phase and have no asthma exacerbations display a specific and distinctive phenotype from those of healthy individuals has yet to be well characterized.

**Objective:**

We aimed to identify the phenotype of basophils from allergic asthmatics in the stable phase and investigate whether such a phenotype is affected by *ex vivo* allergen stimulation.

**Methods:**

We determined by flow cytometry, the expression of surface proteins such as CD25, CD32, CD63, CD69, CD203c, and CD300a and intracellular anti-apoptotic proteins BCL-2, BCL-xL, and MCL-1. We investigated these markers in blood basophils obtained from well-characterized patients with stable-mildly symptomatic form of allergic asthma with no asthma exacerbation and from healthy individuals. Moreover, we determined *ex vivo* CD63, CD69, and CD25 on blood basophils from stable-mildly symptomatic allergic asthmatics upon allergen stimulation.

**Results:**

In contrast to all tested markers, CD25 was significantly increased on circulating basophils in the patient cohort with stable-mildly symptomatic allergic asthma than in healthy controls. The expression levels of CD25 on blood basophils showed a tendency to positively correlate with FeNO levels. Notably, CD25 expression was not affected by *ex vivo* allergen stimulation of blood basophils from stable-mildly symptomatic allergic asthma patients.

**Conclusion:**

Our data identifies CD25 as a unique marker on blood basophils of the stable phase of allergic asthma but not of asthma exacerbation as mimicked by *ex vivo* allergen stimulation.

## 1 Introduction

Basophils are essential effector cells in T2-mediated allergic immune responses. Although basophils account for ≤1% of circulating leukocytes, they actively participate in allergic reactions through the release of effector and immunoregulatory mediators, including vasoactive amines (histamine), lipid metabolites (leukotriene C4, LTC_4_), and T2 cytokines (IL-4 and IL-13). The role of basophils in allergic responses has become more evident with the recognition and in-depth understanding of late-phase responses in chronic allergic inflammatory disorders such as asthma and rhinitis. Several studies in humans and mice identified that basophils infiltrate into lung tissues (1–4). Studies on induced sputum and lung tissues showed that the numbers of basophils in the airways of asthmatics are elevated (1), and they were further increased during asthma exacerbation (2) or following allergen challenge (3). Moreover, high levels of basophil-derived IL-4 in the lung have been detected after segmental allergen challenge (4). These findings thus confirm that basophils are active players of airway inflammation in allergic asthma.

Several studies from others and us reported that an allergic inflammatory milieu, such as IL-3, IL-5, and GM-CSF, transforms basophils into a primed state (5–7). Exposure to these priming stimuli causes increased sensitivity to activation and also enhances multiple biological functions such as inflammatory cytokine release (IL-4, IL-13, IL-8), LTC_4_ formation, chemotaxis, and survival (8). Although other cytokines can induce similar changes, IL-3 is considered the most effective priming factor of human basophils to cause long-term phenotypic and functional changes by itself and in synergy with other stimuli (e.g. FcεRI cross-linking, IL-33) (8–12). For instance, IL-3 boosts the rapid upregulation of CD203c (13) and CD69 (14) on human basophils. Interestingly, prolonged continuous IL-3 receptor-mediated signaling induces the expression of CD25 on human basophils (8).

In addition to its activating effect, IL-3 is also characterized as a pro-survival factor of human basophils. Several studies have indicated that resistance to apoptosis prevails in the allergic airway inflammation of asthma patients (15, 16). Recently, we showed that in the presence of IL-3, basophils are insensitive towards apoptosis induced by IFN-α, extrinsic (TRAIL) (17), and intrinsic (BH3-mimetics) (18) apoptotic stimuli. Furthermore, using BH3-mimetics, we revealed that the observed resistance of basophils to apoptosis in the presence of IL-3 is achieved through the upregulation of anti-apoptotic proteins such as BCL-xL and MCL-1 (19).

Suzuki et al. (20) recently demonstrated that airway basophils show a more activated phenotype than circulating basophils, as evidenced by increased CD203c expression on sputum basophils. Similarly, CD203c was upregulated in blood and sputum basophils after allergen challenge (21) and during asthma exacerbation (13). Thus, basophils undergo several functional and phenotypic alterations during the acute phase of allergic response and asthma exacerbation. Whether such alterations persist during the steady-state of allergic asthma is less well investigated. Hence, in the present study, we investigate whether blood basophils in stable-mildly symptomatic allergic asthma exhibit altered expression levels of surface markers CD25, CD32, CD63, CD69, CD203c, and CD300a, and anti-apoptotic proteins (BCL-2, BCL-xL, and MCL-1) in comparison to blood basophils from healthy controls. Moreover, we study the effect of *ex vivo* allergen challenge on the expression of CD63, CD69, and CD25 on blood basophils from stable-mildly symptomatic allergic asthmatics. Our data confirm that CD25 is a unique marker on human blood basophils in patients with stable or mildly symptomatic of allergic asthma.

## 2 Materials and methods

### 2.1 Study design

Eighteen adults who have a history of stable-mildly symptomatic asthma according to Global Initiative for Asthma (GINA) criteria were recruited from the outpatient clinic of the Department of Pulmonary Medicine, Inselspital, Bern University Hospital, Bern, Switzerland. General inclusion criteria of the study were volunteers of age above 18 years, non-smokers, and all genders were eligible. Allergy was confirmed in this study by a positive skin prick test (SPT) and the detection of specific IgE to aeroallergens. A positive SPT was defined as a wheal of ≥3 mm using the extracts of common aeroallergens such as Olea europea, grass pollen (mixture), birch, hazel, beech, dog and cat epithelia, *Dermatophagoid pteronyssinus*, rye (GREER Laboratories), mugwort, short ragweed, adler, common ash, aspergillus, *Dermatophagoid Farinae*, *Cladosporium herbarum* (if not mentioned all from Bencard AG). The specific IgE to aeroallergens (Sx1: timothy-grass, birch, dog and cat epithelia, *Dermatophagoid pteronyssinus*, rye, mugwort, and *Cladosporium herbarum*) was determined by ImmunoCap technology and regarded as positive if it was ≥0.35 kU/L. Exclusion criteria were infection of airways, use of systemic corticosteroids, and immunosuppressants in the four weeks prior to sample collection. In this study, patients with allergic rhinitis are not included and patients with chronic obstructive pulmonary disease or, allergic bronchopulmonary aspergillosis, or eosinophilic granulomatosis with polyangiitis are excluded as well. The healthy controls (n=10) were non-smokers and reported no history of chronic respiratory disease, and their specific IgE to aeroallergen was <0.35 kU/l. All subjects signed informed consent forms approved by the Ethics Committee of the canton of Bern (no. 2016-01571), and experiments were conducted according to the Declaration of Helsinki.

Included patients (n=18) had 1-2 visits during which blood samples were collected. These blood samples were used to determine counts of eosinophils, neutrophils and basophils using an automated hematology analyzer (Sysmex XS-800i), to analyze surface and internal marker expression in basophils by flow cytometry, and to measure specific IgE against common aeroallergens by ImmunoCap technology. In addition, on every visit, asthma patients were evaluated by spirometry including forced expiratory volume in 1 s (FEV_1_), forced vital capacity (FVC)], asthma control test (ACT) questionnaire score, exhaled nitric oxide fraction (FeNO).

### 2.2 Flow cytometry analysis of basophils in peripheral blood

100µl of EDTA whole blood samples was prepared for flow cytometric measurements using antibody panels as specified in **Table 1**. Surface markers were stained for 15 min at room temperature (RT). Subsequently, red blood cells were lysed using BD Lysing Solution (BD Biosciences) for 15min at RT. Afterward, samples were washed and resuspended in 600µl Staining Buffer A, composed of 1xPBS supplemented with 2% heat-activated FCS and 0.05% sodium azide (Merck Millipore).

**Table 1:** Panels for flow cytometric analysis of blood samples.

	Target	Fluorochrome	Clone
Panel #1	LIN (CD3, CD16, CD19, CD20, CD14, CD56)	FITC	SK7, 3G8, SJ25C1, L27, MfP9, NCAM16.2
	CD123	PerCP-Cy5.5	7G3
	CCR3	Alexa Fluor 647	5E8
	CD203c	PE	97A6
	CD32	BV421	FLi8.26
Panel #2	LIN (CD3, CD16, CD19, CD20, CD14, CD56)	FITC	SK7, 3G8, SJ25C1, L27, MfP9, NCAM16.2
	CCR3	APC-Cy7	5E8
	CD300a	PE	MEM-260
	CD63	V450	H5C6
	CD69	PerCP	FN50
	CD25	APC	BC96
Panel of Intracellular Staining	LIN (CD3, CD16, CD19, CD20, CD14, CD56)	FITC	SK7, 3G8, SJ25C1, L27, MfP9, NCAM16.2
	CD123	PerCP-Cy5.5	7G3
	CCR3	Alexa Fluor 647	5E8
	BCL-2 or BCL-xL or MCL-1	PE	124, 54H6, D2W9E

For intracellular staining, samples were washed with Staining Buffer B, consisting of 1xPBS supplemented with 0.5% BSA and 0.1% sodium azide and permeabilized with Permeabilization solution 2 (BD Biosciences). Next, cells were washed, and intracellular molecules were stained for 30min at RT. Finally, samples were washed and resuspended in 600µl Staining Buffer B. Samples were acquired using BD FACSCanto II. Data were collected using FACSDIVA (all from BD Biosciences) and analyzed by Flowjo software (Treestar Inc). A minimum of 1000 basophils were acquired per sample. The gating strategies used to identify basophils in peripheral blood are described in **Figures S1A–C**.

### 2.3 *Ex vivo* stimulation of basophils

Reagents and protocols of Flow2CAST (Blühmann Laboratories) with slightly modified settings (stimulation time, individual antibodies) were used to activate basophils *ex vivo*. The aeroallergens that elicited the strongest positive response in SPT (birch, *Dermatophagoid pteronyssinus*, *Dermatophagoid farinae*, grass, and rye) in the respective allergic asthma patients were chosen for the *ex vivo* stimulation experiment. Briefly, 50µl of EDTA blood from allergic asthma patients who were sensitive was stimulated with either 50µl (100ng/ml) anti-FcεRI cross-linking antibody 29C6 (Roche), 50µl (20ng/ml or 100ng/ml) of the patient’s respective aeroallergen in 100µl (or 150µl for unstimulated control) stimulation buffer containing IL-3 for 20min at 37°C, 5% CO_2_.

Cells were stained simultaneously with anti-CCR3 Alexa Fluor 647 (5E8; BioLegend), anti-CD63 V450 (H5C6; BD Biosciences), anti-CD69 PerCP (FN50; BioLegend), anti-CD25 APC (BC96; BioLegend). Data were acquired using FACSCanto with FACS Diva software. For gating strategy, debris and doublets were excluded first, followed by gating for SSC^low^/CCR3^pos^ basophils. Within SSC^low^/CCR3^pos^ basophils, the cells were further gated for CD63^pos^/CCR3^pos^ and CD63^neg^/CCR3^pos^ for degranulated and non-degranulated basophils, respectively as shown in **Figure S1D**. CD69 and CD25 expression was subsequently analyzed in CD63^neg^ and CD63^pos^ basophils, respectively.

### 2.4 Statistical analysis

Data were analyzed using GraphPad Prism 8.3 (GraphPad). The alpha level was set to 0.05. If not mentioned, otherwise data are represented as mean ± SEM. Statistical correlations were evaluated by the Spearman rank test. For two-group comparisons, two-tailed Student t-test, and Mann-Whitney test were used. One-way ANOVA with Dunnett`s or Bonferroni`s multiple comparison test was used when more than two groups were analyzed. Results were considered significant if P values were <0.05. P values were defined as **** P< 0.0001, *** P<0.001, ** P<0.01 and * P<0.05. ns, not significant.

## 3 Results

### 3.1 Patient characteristics

Demographic and clinical characteristics of the recruited subjects are summarized in **Table 2** and **Supplementary Table 1**. The mean age of the study participants was 37 years (range: 18-59 years), and 72% of them were female. All participants had a history of allergic asthma and were tested positive for at least one among the 16 common aeroallergens (skin prick test, wheal ≥3 mm and ImmunoCap sx1>0.35kU/L). Only one out of eighteen patients showed non-detectable specific IgE (ImmunoCap sx1<0.35kU/L) during one of the visits (**Supplementary Table 2**). No subject reported or had documented asthma exacerbations during the study period. The asthma control test (ACT) scores (20.97 ± 3.537, mean ± SD) showed that the patients enrolled were suffering from a stable-mildly symptomatic form of allergic asthma. The cut-off for ACT scores used were 20-25: stable, 16-19: mild and < 15: severe. Asthma was not under control for three out of eighteen patients (for three patients in the first visit and for one patient for every visits) (**Supplementary Table 1**). The fractional exhaled nitric oxide (FeNO) values (28.28 ± 26.89, mean ± SD) indicated that the majority of the sample size (90%) did not associate with severe/chronic lung inflammation (FeNO<50ppb, **Supplementary Table 1**). Altogether, the ACT scores and FeNO values indicated that the subjects enrolled in this study had stable-mildly symptomatic form of allergic asthma during the majority of visits (**Supplementary Table 1**).

**Table 2:** Characteristics of the patient cohort.

Characteristics	Values	Sample size
Age, yrs		18
Mean (Range)	37.36 (18-59)	
Gender, n		18
Female	13	
Male	5	
BMI, kg/m^2^		18
Mean ± SD	24.83 ± 4.524	
% Atopy (skin prick test)*	100	18
Atopy (specific IgE), kU/L§		18
Mean ± SD	24.96 ± 26.87	
ACT score		18
Mean ± SD	20.97 ± 3.537	
FeNO, ppb		18
Mean ± SD	28.28 ± 26.89	
Blood basophils, G/L		18
Mean ± SD	0.042 ± 0.015	
Blood eosinophils, G/L		18
Mean ± SD	0.246 ± 0.183	
Blood neutrophils, G/L		18
Mean ± SD	3.262 ± 1.22	
% FEV_1_ predicted		18
Mean ± SD	91.83 ± 13.92	
% FVC predicted		18
Mean ± SD	100.8 ± 11.81	

BMI, body mass index; FeNO, fractional exhaled nitric oxide; ppb, parts per billion; ACT, asthma control test; G/L, 10^6^ cells/L; FEV1, forced expiratory volume in 1 s; FVC, forced vital capacity; Mean ± SD, mean± Standard deviation (For each clinical parameter, the mean value for each individual during their 1-2 visits was first calculated, and then the mean and standard deviation of these mean values were determined).

*Atopy on skin prick test was defined by at least one positive test (wheal ≥3 mm) against common aeroallergens such as Olea europea, grass pollen (mixture), birch, hazel, beech, dog and cat epithelia, *Dermatophagoid pteronyssinus*, rye, mugwort, short ragweed, adler, common ash, aspergillus, *Dermatophagoid farinae*, *Cladosporium herbarum*.

^§^At least one of the IgE RAST ≥ 0.35 kU/L (Dermatophagoides pteronyssinis, cat and dog dander, timothy, rye, Cladosporium herbarum, birch, mugwort).

### 3.2 Unique upregulation of CD25 expression on blood basophils in patients with stable-mildly symptomatic form of allergic asthma

**Figure 1:**
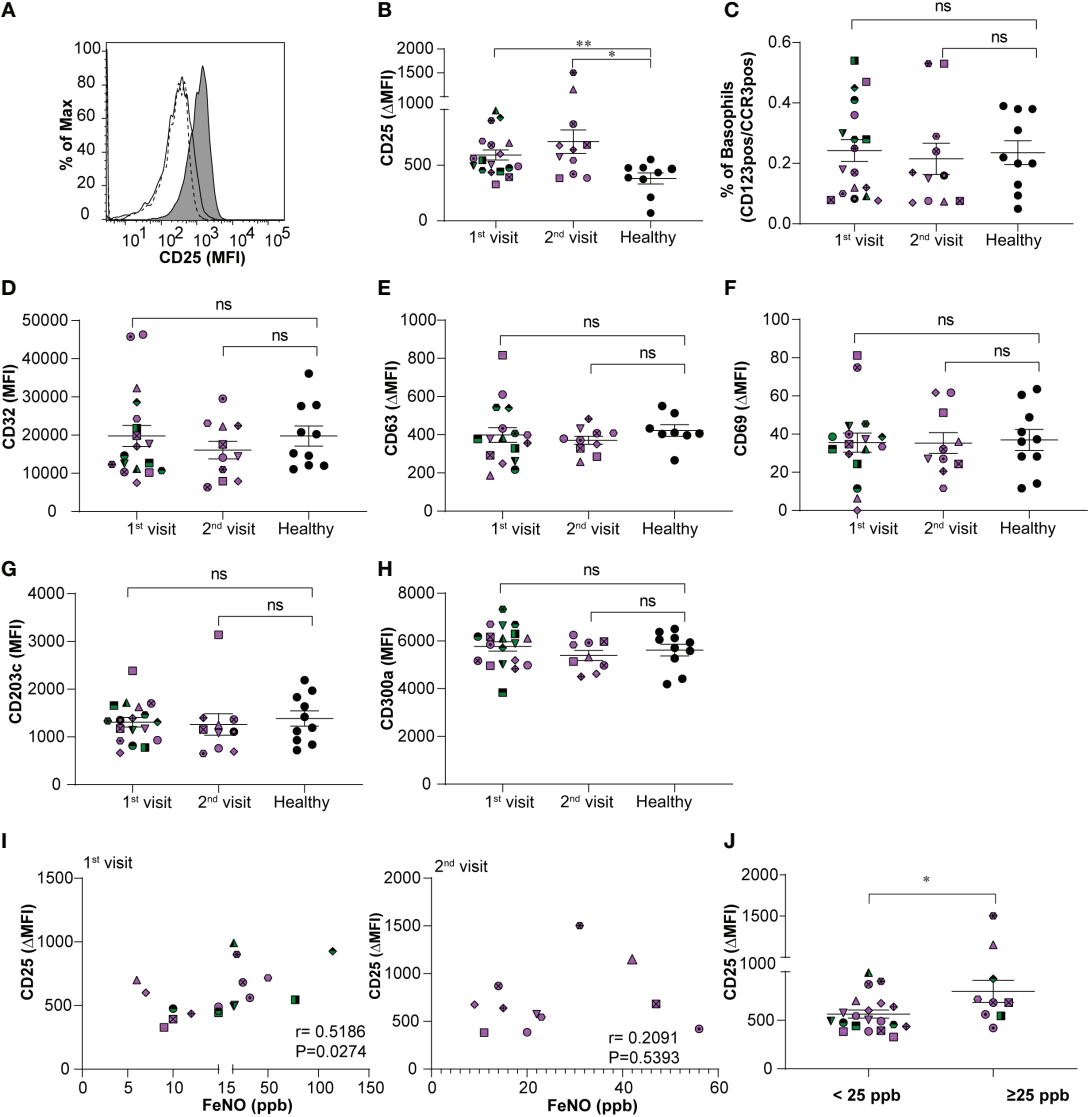
Relationship between expression levels of CD25 on basophils and FeNO levels in stable-mildly symptomatic allergic asthma patients. **(A)** Representative histogram showing CD25 expression level (MFI) on blood basophils in stable-mildly symptomatic allergic asthma patient (gray area) compared to healthy control (solid line) and FMO control (dotted line). **(B, D-H)** The expression levels of surface markers on blood basophils of stable-mildly symptomatic allergic asthma patients for the first (n=18) and second visit (n ≤ 11) compared to healthy controls (n≥9) measured by flow cytometry. Data are represented as mean ± SEM and reported as ΔMFI (MFI of test-MFI of FMO control) for CD25, CD63, CD69 and as MFI for CD32, CD203c and CD300a. Results were analyzed by two-tailed unpaired Mann-Whitney test.**P=0.0065, *P=0.0159, ns=non-significant **(C)** The relative percentage of blood basophils (dual positive for CD123 and CCR3) from stable-mildly symptomatic allergic asthma patients during their first (n=18) and second (n=11) visits compared to healthy subjects (n=10) **(I)** Correlation between CD25 expression levels (ΔMFI) and FeNO levels (ppb) in stable-mildly symptomatic allergic asthma patients of the first (left, n=18) and second visit (right, n=11). Spearman`s rank test was used for correlation analysis. Spearman coefficient (r) and level of significance (P) are indicated within the graph. **(J)** Surface expression levels of CD25 (ΔMFI) on blood basophils between <25 ppb (n=20) and ≥25 ppb FeNO level (n= 9) groups within stable-mildly symptomatic allergic asthma patients. Data are represented as mean ± SEM and analyzed using two-tailed unpaired Mann-Whitney test. *P<0.05. **(B-J)** Every patient is represented with specific symbol, while healthy controls are shown in closed black symbols. Patients who had both 1^st^ and 2^nd^ visits are marked in purple and those who had only 1^st^ visit are marked in green color. MFI, median fluorescence intensity; FMO, fluorescence minus one; Healthy, healthy control subjects.

During asthma exacerbation, blood basophils display an activated phenotype (13). Moreover, anti-apoptotic conditions prevail in basophils under allergic conditions (17, 19). Whether the activated and anti-apoptotic phenotype persist during the steady-state of stable allergic asthma is less well investigated. Hence, we compared the immunophenotype of basophils and their predisposition to apoptosis in the stable-mildly symptomatic allergic asthma patient cohort and healthy controls. As shown in **Figures 1A, B**, flow cytometry analysis revealed that CD25 expression on blood basophils was significantly higher (P=0.0065 and P=0.0159) for the first and second visit, respectively in stable-mildly symptomatic allergic asthma patients than in healthy control subjects. The proportion of basophils from our patient cohort and healthy controls were comparable (**Figure 1C**). There was no significant difference in CD25 expression when comparing the first and second visit of the stable-mildly symptomatic allergic asthma patients. For all other analyzed markers, particularly, the activation markers CD63, CD69, CD203c and CD300a no significant difference was observed (**Figures 1D–H** and **Figures S2A-C**). This observation indicates that circulating basophils from stable-mildly symptomatic asthmatics were not hyperactivated.

Correlation analysis between CD25 expression on blood basophils and absolute counts (G/L) of eosinophils, basophils and neutrophils, respectively, revealed no significance (**Figures S3A-C**). FeNO levels of stable-mildly symptomatic allergic asthmatics showed a weak positive correlation (r=0.5186; P=0.0274) when comparing the CD25 expression levels on circulating basophils of the first visit (**Figure 1I**, left), but not of the second visit (r=0.2091; P=0.5393) (**Figure 1I**, right). This inconsistent observation in the correlation between CD25 and FeNO in the first and second visits could be due to the lower sample number in the second visit (n=11) than in the first visit (n=18). The levels of CD25 (P=0.03) were significantly higher expressed in patients with intermediate FeNO values (FeNO ≥25 ppb; **Figure 1J**) according to American Thoracic Society guidelines (22) than in patients with low FeNO (FeNO <25 ppb).

### 3.3 CD25 expression on basophils is not affected by allergen challenge *ex vivo*

**Figure 2:**
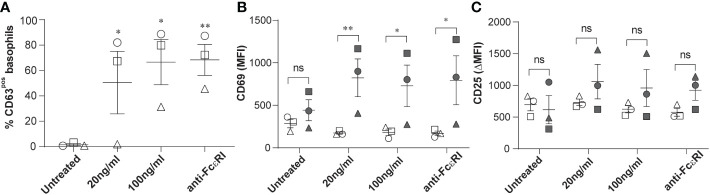
Effects of *ex vivo* allergen challenge on CD63, CD69, and CD25 expression in activated and non-activated basophils. Whole blood samples from stable-mildly symptomatic allergic asthma patients (n=3) were stimulated with their respective allergen (20ng/ml or 100 ng/ml) or anti-FcεRI cross-linking antibody 29C6 (100 ng/ml). Unstimulated controls were exposed to stimulation buffer only. **(A)** Percentage of CD63^pos^ basophils in unstimulated controls, 20 ng/ml, and 100 ng/ml allergen and anti-FcεRI cross-linking antibody stimulated samples. **(B, C)** MFI of CD69 **(B)** and ΔMFI (MFI of test-MFI of FMO control) of CD25 **(C)** are shown on CD63^neg^ (open symbols) and CD63^pos^ basophils (closed symbols). **(A-C)** Patients 1, 2, and 3 are represented as circle, square, and triangle symbols, respectively. Data are shown as mean ± SEM and analyzed using one-way ordinary ANOVA tests, with Dunnett`s **(A)** or Bonferroni`s **(B, C)** multiple group comparison test. **P<0.01 and *P<0.05. ns, not significant.

The surface expression of CD63, CD203c and CD69 is commonly used to assess human basophil activation upon acute hypersensitivity allergic reaction. This encouraged us to investigate further whether the expression levels of CD25 on circulating basophils of patients with stable-mildly symptomatic allergic asthma are altered upon *ex vivo* allergen exposure. We confirmed basophil degranulation by a significant increase in the surface expression of CD63 in peripheral basophils, and the increment of expression was found to be dependent on allergen concentration, as shown in **Figure 2A**. In line with previous studies, CD69 expression was significantly upregulated in CD63pos basophils compared to CD63neg basophils (**Figure 2B**). Interestingly, the *ex vivo* stimulation using both allergen and anti-FcϵRI cross-linking antibody did not affect CD25 expression (**Figure 2C**). In summary, our data show that the level of CD25 expression is increased in stable-mildly symptomatic allergic asthma but is not affected by *ex vivo* allergen stimulation.

## 4 Discussion

There is growing evidence on the important functions of basophils in T2-driven inflammation in asthma. Our current study extends this knowledge by investigating the expression levels of multiple surface and intracellular markers of basophils, which indicate their effector and immunoregulatory function in allergic asthma. This is the first report on identifying high levels of spontaneous CD25 expression in circulating basophils in stable-mildly symptomatic allergic asthmatics compared to healthy subjects.

Human basophils express CD25 under physiologic conditions. Its expression at both mRNA and protein levels can be significantly enhanced in the presence of IL-3 stimulation (8, 23). In our previous study, we observed that a continuous IL-3 exposure for up to 5 days was needed to induce CD25 expression. Thus, CD25 can be used as an activation marker to identify late IL-3 priming of basophils. By combining our previous *in vitro* data and current *in vivo* observations, we speculate that circulating basophils from stable asthma patients are in continuous and prolonged exposure to IL-3, secreted by activated T cells, mast cells, or basophils (24), ultimately resulting in the upregulation of CD25. However, as an evidence, the measurement of IL-3 in the sera from the mildly-stable symptomatic allergic asthmatics will be necessary. Nevertheless, we cannot rule out the effects of other cytokines on the expression of CD25 in basophils. Although human basophils express CD25 under physiological conditions, there is only limited knowledge regarding its functional significance in human basophils. A report by Zhao et al. recently showed that IL-2 binds to the CD25 receptor of human basophils, resulting in induced expression of inflammatory cytokines like IL-5 and GM-CSF (23). Interestingly, IL-5 and GM-CSF are also crucial for eosinophil infiltration into the target tissue of allergic inflammation. Thus, such an “*in vivo* IL-3 or IL-2-primed” effect of basophils may further recruit peripheral eosinophils into the airways. Furthermore, in human eosinophils, IL-2 induces enhanced release of eosinophil cationic protein from CD25 expressing but not from CD25 negative eosinophils (25).

In contrast to other reports (13), we did not observe any significant changes in the surface markers such as CD203c and CD63 in our cohort of stable-mildly symptomatic allergic asthma patients. We presume that this difference in the results may be due to the difference in the study cohort, as upregulation of CD203c and CD63 in blood basophils was observed in asthma patients with exacerbation, but not in patients having stable phase of allergic asthma. Furthermore, we stimulated blood basophils with allergen *ex vivo* to mimic asthma exacerbation after allergen challenge. In contrast to the increased CD63 and CD69 levels, we observed no change in the expression levels of CD25 on degranulated, CD63-positive basophils, upon *ex vivo* allergen stimulation. Furthermore, although we found a significant higher expression of CD25 in stable-mildly symptomatic asthmatics having FeNO >25ppb compared to patients showing FeNO<25ppb, the correlation of FeNO and CD25 was not consistent between the visits. Altogether, these results suggest that CD25 is not a marker to monitor the asthma exacerbation after allergen challenge, but CD25 can be used as a unique marker for stable-mildly symptomatic allergic asthma patients.

Daclizumab, a humanized monoclonal antibody against CD25, improved pulmonary function and asthma control in moderate to severe asthma patients by IL-2R blockade in activated T cells (26). Its mode of action may also extend to other CD25-expressing effector cells such as basophils and eosinophils. Further studies are warranted to examine the impact of such treatments on basophils *in vivo* functions.

We identified CD25 as a novel biomarker for late *in vivo* priming of human basophils in stable-mildly symptomatic allergic asthma. Our findings highlight the importance of basophils in the pathogenesis of allergic asthma. Further studies are required to investigate the underlying mechanisms and the efficacy of new CD25 and/or basophil targeted therapies.

## Data availability statement

The raw data supporting the conclusions of this article will be made available by the authors, without undue reservation.

## Ethics statement

All subjects signed informed consent forms approved by the Ethics Committee of the canton of Bern (no. 2016-01571), and experiments were conducted according to the Declaration of Helsinki. The patients/participants provided their written informed consent to participate in this study.

## Author contributions

JI, LR, SB conducted the experiments, and JI, LR, and MF analyzed data. JI prepared the figures. NP and CG recruited the patients. TH performed FeNO measurements, and the skin prick test. MF designed and supervised the study. JI and MF wrote the manuscript and all the aforementioned authors discussed the manuscript. All authors contributed to the article and approved the submitted version.
