# Supplementary material for: CD25 as a unique marker on human basophils in stable-mildly symptomatic allergic asthma
Source: Front Immunol. 2023 Jan 5;13:1031268. doi: 10.3389/fimmu.2022.1031268 (PMC9849741; doi:10.3389/fimmu.2022.1031268)
Supplement: Supplementary file 1 [file DataSheet_1.docx]

Supplementary Material

# Supplementary Figures

## Supplementary Figure 1


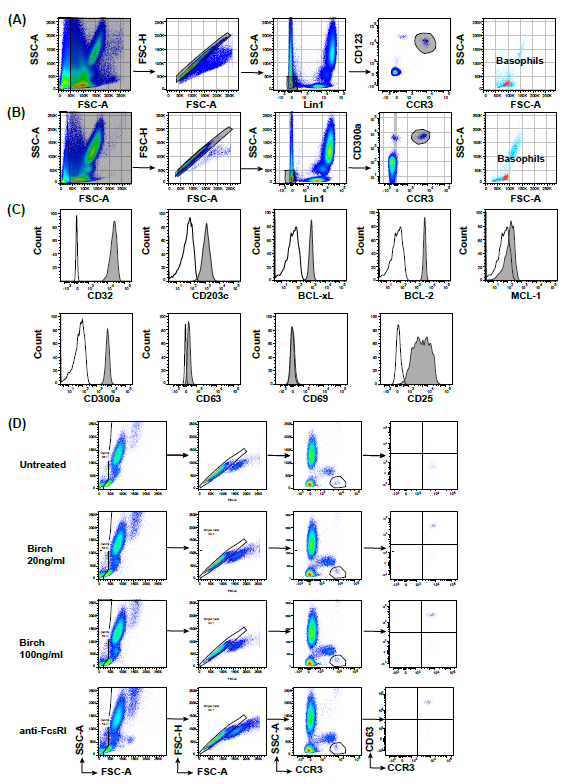


**Supplementary figure 1: Gating strategy for identifying basophils in peripheral blood.**

(**A**-**B**) To identify blood basophils, debris and doublets were excluded first. This was followed by gating for SSC^low^/Lin1^neg^ cells. Within SSC^low^/Lin1^neg^ population, basophils were identified based on the dual expression of CD123/CCR3 (**A**) and CD300a/CCR3 (**B**), respectively. (**C**) Representative histograms of fully stained blood samples (grey histogram) with respect to FMO or unstained samples (open histogram) are shown. (**A**&**B**) Back gating of the basophil population (red dots) is depicted on top of all cells that are negative of debris (blue dots). (**D**) Blood basophils after *ex vivo* stimulation were identified by excluding the debris and doublets, followed by gating for SSClow/CCR3pos basophils. Within SSClow/CCR3pos basophils, the cells were further gated for CD63pos/CCR3pos and CD63neg/CCR3pos for degranulated and non-degranulated basophils, respectively. Representative dot plots of a patient blood sample stimulated with birch allergen (20ng/ml or 100ng/ml) compared to the negative (untreated) and positive (anti-FcεRI cross-linking antibody 29C6) control.

## Supplementary figure 2


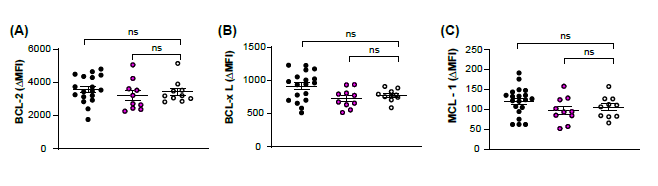


**Supplementary figure 2: Comparison of the expression levels of intracellular anti-apoptotic proteins on blood basophils from stable-mildly symptomatic allergic asthma patients during their first and second visits and healthy controls.**

The expression levels of anti-apoptotic proteins (**A**-**C**) on blood basophils of stable-mildly symptomatic allergic asthma patients during their first (n=18; black closed symbols) and second (n≤11; purple closed symbols) visits and healthy controls (n≥9; opened symbols) were determined by flow cytometry. Data are represented as mean±SEM, reported as ΔMFI and analyzed by two-tailed unpaired Mann-Whitney test. ΔMFI, MFI of test MFI of FMO control. ns, not significant; Healthy, healthy subjects.

## Supplementary figure 3


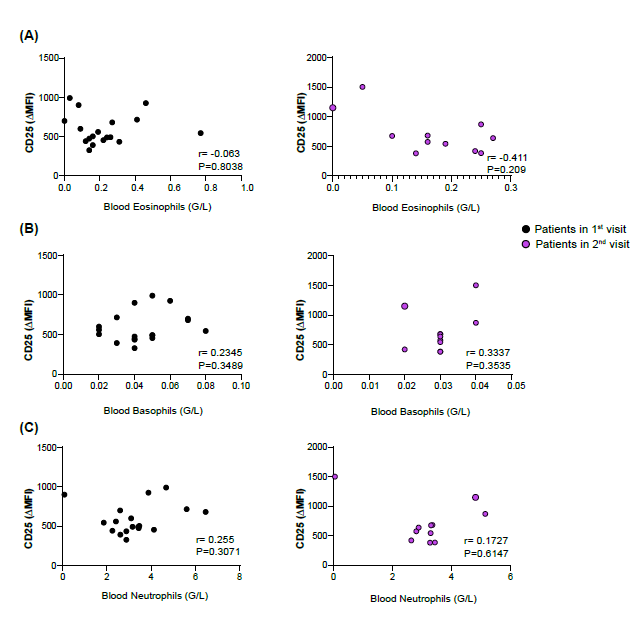


**Supplementary figure 3: Correlation between CD25 expression levels (ΔMFI) on circulating basophils and the absolute counts of blood eosinophils, basophils and neutrophils in stable-mildly symptomatic allergic asthma patients.**

The correlation between CD25 expression levels on blood basophils and absolute counts of eosinophils (**A**), basophils (**B**), and neutrophils (**C**) in the blood of stable-mildly symptomatic allergic asthma patients of the first (left, n=18, black symbols) and second visit (right, n=11, purple symbols). Spearman`s rank test was used for correlation analysis. Spearman coefficient (r) and level of significance (P) are indicated within the graph. ΔMFI, MFI of test MFI of FMO control; G/L, 10^6^ cells/L.

# Supplementary Tables

## Supplementary Table 1

| Patient ID | ACT score | | FeNO (ppb) | | Blood Eosinophils (G/L) | | Blood Basophils (G/L) | | Blood Neutrophils (G/L) | |
| --- | --- | --- | --- | --- | --- | --- | --- | --- | --- | --- |
|  | 1st visit | 2nd visit | 1st visit | 2nd visit | 1st visit | 2nd visit | 1st visit | 2nd visit | 1st visit | 2nd visit |
| P001 | 13 | 25 | 10 | 47 | 0.17 | 0.16 | 0.02 | 0.03 | 2.61 | 3.36 |
| P002 | 18 | 20 | 25 | 14 | 0.27 | 0.25 | 0.07 | 0.04 | 6.47 | 5.15 |
| P003 | 22 | 16 | 15 | 20 | 0.24 | 0.25 | 0.05 | 0.03 | 3.42 | 3.44 |
| P004 | 10 | 14 | 17 | 22 | 0.16 | 0.16 | 0.02 | 0.03 | 3.47 | 2.81 |
| P005 | 24 | 25 | 9 | 11 | 0.14 | 0.14 | 0.04 | 0.03 | 2.88 | 3.28 |
| P006 | 22 | 23 | 6 | 42 | 0 | 0 | 0.07 | 0.02 | 2.6 | 4.82 |
| P007 | 23 | 20 | 7 | 9 | 0.09 | 0.1 | 0.02 | 0.03 | 3.1 | 3.31 |
| P008 | 9 | 19 | 12 | 15 | 0.31 | 0.27 | 0.04 | 0.03 | 2.88 | 2.89 |
| P009 | 23 |  | 10 |  | 0.14 |  | 0.04 |  | 3.44 |  |
| P010 | 23 |  | 15 |  | 0.22 |  | 0.05 |  | 4.13 |  |
| P011 | 22 | 24 | 32 | 56 | 0.19 | 0.24 | 0.02 | 0.02 | 2.41 | 2.64 |
| P012 | 20 | 20 | 50 | 23 | 0.41 | 0.19 | 0.03 | 0.03 | 5.61 | 3.3 |
| P013 | 20 |  | 15 |  | 0.12 |  | 0.04 |  | 2.25 |  |
| P014 | 25 | 25 | 19 | 31 | 0.08 | 0.05 | 0.04 | 0.04 | 0.08 | 0.05 |
| P015 | 24 |  | 114 |  | 0.46 |  | 0.06 |  | 3.88 |  |
| P016 | 22 |  | 16 |  | 0.52 |  | 0.06 |  | 4.68 |  |
| P017 | 21 |  | 16 |  | 0.26 |  | 0.05 |  | 3.16 |  |
| P018 | 25 |  | 77 |  | 0.77 |  | 0.08 |  | 1.86 |  |

ACT, asthma control test; FeNO, fractional exhaled nitric oxide; ppb, parts per billion; G/L, 10^6^ cells/L.

§ The cut-off for ACT scores used were 20-25: stable, 16-19: mild and < 15: severe

## Supplementary Table 2
